# Human xylosyltransferases – mediators of arthrofibrosis? New pathomechanistic insights into arthrofibrotic remodeling after knee replacement therapy

**DOI:** 10.1038/srep12537

**Published:** 2015-07-28

**Authors:** Isabel Faust, Philipp Traut, Frank Nolting, Jan Petschallies, Elena Neumann, Elke Kunisch, Joachim Kuhn, Cornelius Knabbe, Doris Hendig

**Affiliations:** 1 Institut für Laboratoriums- und Transfusionsmedizin, Herz- und Diabeteszentrum Nordrhein-Westfalen, Universitätsklinik der Ruhr-Universität Bochum, Bad Oeynhausen, Germany; 2 Klinik am Rosengarten, Bad Oeynhausen, Germany; 3 Orthopaedicum Hannover, Zentrum für orthopädische Chirurgie, Hannover, Germany; 4 Justus-Liebig Universität Gießen, Internal Medicine and Rheumatology, Kerckhoff-Klinik Bad Nauheim, Bad Nauheim, Germany; 5 Universitätsklinikum Jena, Rudolf-Elle Waldkrankenhaus Eisenberg, Eisenberg, Germany

## Abstract

Total knee replacement (TKR) is a common therapeutic option to restore joint functionality in chronic inflammatory joint diseases. Subsequent arthrofibrotic remodeling occurs in 10%, but the underlying pathomechanisms remain unclear. We evaluated the association of xylosyltransferases (XT), fibrotic mediators catalyzing glycosaminoglycan biosynthesis, leading to arthrofibrosis as well as the feasibility of using serum XT activity as a diagnostic marker. For this purpose, synovial fibroblasts (SF) were isolated from arthrofibrotic and control synovial biopsies. Basal α-smooth muscle actin expression revealed a high fibroblast-myofibroblast transition rate in arthrofibrotic fibroblasts. Fibrotic remodeling marked by enhanced XT activity, α-SMA protein expression as well as xylosyltransferase-I, collagen type III-alpha-1 and *ACTA2* mRNA expression was stronger in arthrofibrotic than in control fibroblasts treated with transforming growth factor-β1 (TGF-β1). Otherwise, no differences between serum levels of XT-I activity or common fibrosis markers (galectin-3 and growth differentiation factor-15 levels (GDF-15)) were found between 95 patients with arthrofibrosis and 132 controls after TKR. In summary, XT-I was initially investigated as a key cellular mediator of arthrofibrosis and a target for therapeutic intervention. However, the blood-synovial-barrier makes arthrofibrotic molecular changes undetectable in serum. Future studies on monitoring or preventing arthrofibrotic remodeling should therefore rely on local instead of systemic parameters.

If current medical therapies are exhausted, knee replacement surgery (TKR) can restore quality of life in patients with degenerative joint disorders. In patients suffering from osteoarthritis (OA) or rheumatoid arthritis (RA), the benefit of TKR has been proven^1^. Nevertheless, the durability of endoprosthetic implants is limited: particle-induced loosening, infection, and arthrofibrosis can shorten implant durability. Histopathologically, four types of neosynovitis/periprosthetic membrane were identified^2^. Primary arthrofibrosis is defined as painful impairment of joint flexibility due to fibrotic tissue remodeling after joint trauma or surgery. This entity needs to be distinguished from secondary arthrofibrosis, a condition attributable to inaccurate implant positioning^3^. Although the predicted incidence of primary arthrofibrosis after knee replacement is 10%, its pathogenesis is mostly unclear and no diagnostic marker has been described^4^. Until today, the diagnosis is based on histopathological findings and clinical symptoms such as loss of motion^5^,^6^. Treatment of arthrofibrosis is challenging and several therapeutic strategies have been discussed^4^,^7^.

The reparative inflammatory mechanism in arthrofibrosis seems to resemble that of other fibrotic disorders. It has been well, but not completely characterized^8^. Increased secretion of profibrotic molecules such as transforming growth factor-β1 (TGF-β1), the resultant transformation of resident fibroblasts to activated myofibroblasts as well as matrix accumulation and stiffening are accepted as key steps in the fibrotic process. In fibrosis, activated myofibroblasts do not initiate apoptosis after wound healing, but continue to synthesize matrix components, thereby contributing to pathological scar formation. The imbalance between extracellular matrix (ECM) synthesis and degradation gives rise to an excessive accumulation of ECM molecules such as collagens and proteoglycans in the intercellular space^9^,^10^.

Proteoglycans consist of a core-protein covalently linked to glycosaminoglycan chains. Glycosami-noglycans regulate important cellular functions including proliferation, sequestration, and release of growth factors and maintain cartilage hydration^11^,^12^. Glycosaminoglycan biosynthesis is initiated by xylosyltransferase-I and -II (XT-I/-II, EC 2.4.2.26), both Golgi-resident isoenzymes catalyzing the rate-limiting step in proteoglycan glycosylation. Since XT are secreted into the ECM with the xylosylated acceptor proteoglycan, quantification of serum XT activity provides a powerful technique for monitoring dysregulated tissue remodeling processes^13^,^14^. Upregulation of XT activity in serum as well as increased cellular *XYLT1* expression are correlated with disorders of proteoglycan accumulation, e.g. scleroderma and liver fibrosis^15^,^16^. Furthermore, it could be demonstrated that *XYLT1* expression is induced by TGF-β1 in the early onset of OA cartilage repair, while *XYLT1* expression is reduced by IL-1β in late stage OA. Therefore, XT is a central regulator of cartilage destruction, cartilage repair, and glycosaminoglycan homeostasis in fibrosis and degenerative joint diseases^17^,^18^.

This study aimed to investigate the possible relationship between the development of arthrofibrosis after knee replacement surgery and changes in XT expression and activity. For this purpose, we combined experimental approaches with a potential to elucidate the complex regulatory processes in arthrofibrosis. In summary, we demonstrated for the first time that XT-I is not only a key regulator of arthrofibrotic remodeling in synovial fibroblasts (SF) *in vitro*, but also a probable target for therapeutic intervention.

## Material and Methods

### Patients and controls

The study cohort comprised 95 patients with arthrofibrosis and 132 controls. All underwent total knee replacement (TKR) because of functional impairment due to arthritic degeneration or posttraumatic arthropathy. The proportion of revision surgeries was almost equal in the cohorts of controls and arthrofibrosis patients (controls: 24.2%; arthrofibrosis patients: 33.7%). The period of time from TKR to the beginning of orthopedic rehabilitation and diagnosis within this study (Time_diagnosis_) depended on the group and the clinical course of normal healing or arthrofibrotic development. Thus, blood sampling and group classification were performed in the rehabilitation center after TKR and immediately before commencing the rehabilitation program. At this time, the diagnosis of arthrofibrosis was established. Clinical patient characteristics are summarized in Table 1.

In accordance with previous studies, arthrofibrosis in study patients as well as tissue donors was diagnosed based on clinical findings such as synovial hyperplasia, reduced range of motion and ongoing knee pain. We used the disease classification provided by the World Health Organization (ICD-10, version 2012). While secondary arthrofibrosis and implant malpositioning were excluded by X-ray examination, postoperative complications such as infection or deep vein thrombosis were excluded as described before^4^,^6^,^19^. Patients with actual or suspected arthrofibrosis were divided into three groups due to diagnostic confidence: arthrofibrosis score 1 (Afib1): suspected arthrofibrosis based on clinical findings, short Time_diagnosis_ and incomplete differential diagnosis, Afib2: arthrofibrosis based on clinical findings, Afib3: arthrofibrosis based on strong clinical findings as well as histopathological findings reported by an external institution.

Platelet counts were measured with the Cell-Dyn Ruby (Abbott, Illinois, USA). Biopsies were obtained from patients undergoing knee revision surgery. All serum and tissue samples were collected by the authors in accordance with the German Act on Medical Devices (MPG, guideline 98/79/EG) for the collection of human residual material to evaluate suitability of an *in vitro* diagnostic medical device (§24). The need for informed consent and ethical approval was waived since all materials used were (surgical) waste from routine laboratory diagnostics and knee replacement surgery.

### Preparation and cultivation of synovial fibroblasts (SF)

Synovial biopsies from controls and from the Afib2 group were decontaminated in 70% ethanol and washed with PBS (Invitrogen, San Diego, USA). Minced pieces were digested at 37 °C with 0.1% trypsin for 1 h and 0.1% collagenase for 3 h. Cells were cultured routinely in DMEM (Invitrogen, San Diego, USA) supplemented with 10% FCS (Pan biotech, Aidenbach, Germany), 1% antibiotic/antimycotic solution (100x; PAA, Pasching, Austria) and 2% L-glutamine (200 mM; PAA, Pasching, Austria) under a humidified atmosphere of 5% CO_2_ at 37 °C. Outgrowing cells were cultured and split after reaching confluence. SF from control and Afib patients were checked for expression of CD90 by immunofluorescence analysis and used at passages four to eleven.

### SF induction with TGF-β1

When reaching confluence, SF were trypsinized and seeded (40 cells/mm^2^) in triplicate for biological samples. After 24 h, a serum withdrawal of 10 to 0.1% FCS was performed over 24 h. SF were treated with TGF-β1 (5 ng/mL, Miltenyi Biotech, Bergisch Gladbach, Germany) or vehicle for 48 h. Cell lysates were resuspended in lysis buffer (Macherey-Nagel, Düren, Germany) and stored at −80 °C. Cell culture supernatants were stored at −20 °C.

### Immunohistochemistry

SF seeding (40 cells/mm^2^), serum withdrawal, and cell treatment with TGF-β1 were performed as described before^20^. After 120 h, cells were washed with PBS and fixed in acetone/methanol for 10 min. After two washing steps, blocking in 1% bovine serum albumin was performed for 1 h. After an additional washing step, cells were incubated with the primary monoclonal mouse anti-human smooth muscle actin antibody Clone 1A4 (1:50; Dako, Hamburg, Germany) for 2 h. Excess antibodies were removed by two washing steps, followed by incubation with monoclonal FITC conjugated goat-anti-mouse IgG/IgM (H + L) secondary antibody (1:100, Code Number 115-095-068; Dianova, Hamburg, Germany) for 1 h. Finally, cells were washed and covered with PBS. Immunofluorescence signals were visualized and photographed with the Eclipse TE2000-S microscope (Nikon, Düsseldorf, Germany). Corrected total cell fluorescence was compared between groups using Image J.

### Nucleic acid extraction

Total RNA extraction was performed as previously described^20^. DNA extraction was completed with the DNA Spin Blood Kit (Macherey-Nagel, Düren, Germany). Nucleic acid concentrations were determined photometrically using the NanoDrop 2000 (Peqlab, Erlangen, Germany).

### Reverse transcription and quantitative real-time PCR

1 μg RNA was reverse transcribed to cDNA by using SuperScript II RT (Invitrogen, San Diego, USA). cDNA was used to analyze mRNA expression levels by quantitative real-time PCR as described before^20^. The mRNA expression was analyzed using intron-spanning primers (Table 2) for housekeeping genes (hypoxanthine phosphoribosyltransferase 1 (*HPRT*), glyceraldehyde-3-phosphate dehydrogenase (*GAPDH*), β2-microglobuline (*B2M*)), as well as xylosyltransferases (*XYLT1, XYLT2*), alpha smooth muscle actin-2 (*ACTA2*), and collagens (*COL1A1, COL3A1, COL5A*). Relative transcription levels were verified in triplicate and calculated by the delta-delta Ct-method considering PCR efficiency^21^. The normalization factor calculation was based on the geometric mean of the expression levels of *HPRT*, *GAPDH* and *B2M*.

### XT activity assays

Quantification of XT activity in serum and cell culture supernatants of SF was determined as described previously. The radiochemical method is based on the incorporation of [^14^C]D-xylose (Du Pont, Homburg, Germany) into silk fibroin receptor protein. Disintegrations per minute (dpm) were measured in duplicate for every sample and, in case of activity determination in cell culture supernatants, referenced to the total DNA concentration of the appropriate cell lysate^22^. The HPLC electrospray ionization tandem mass spectrometry method used a synthetic peptide Biotin-NH-QEEEGSGGGQKK(fluorescein)-CONH_2_ as acceptor protein and was described in the past^23^.

### Galectin-3 and GDF-15 ELISA

Galectin-3 and GDF-15 (growth differentiation factor-15) levels were determined in serum samples using commercially available kits (R&D systems, Minneapolis, USA).

### Statistics

Relative mRNA expression levels and XT activities in SF are shown as mean ± 95% CI. Serum concentrations are illustrated as box and whisker plots (5^th^ to 95^th^ percentile). Experimental data were analyzed with the nonparametric two-tailed Mann-Whitney U-Test using GraphPad Prism 5.0. Spearman’s test was applied for nonparametric correlation of dependencies between XT activity and platelet count or ROM, respectively. p values less than 0.05 were considered statistically significant.

## Results

### Effects of TGF-β1 stimulation on XYLT and ECM mRNA expression in SF

**Figure 1:**
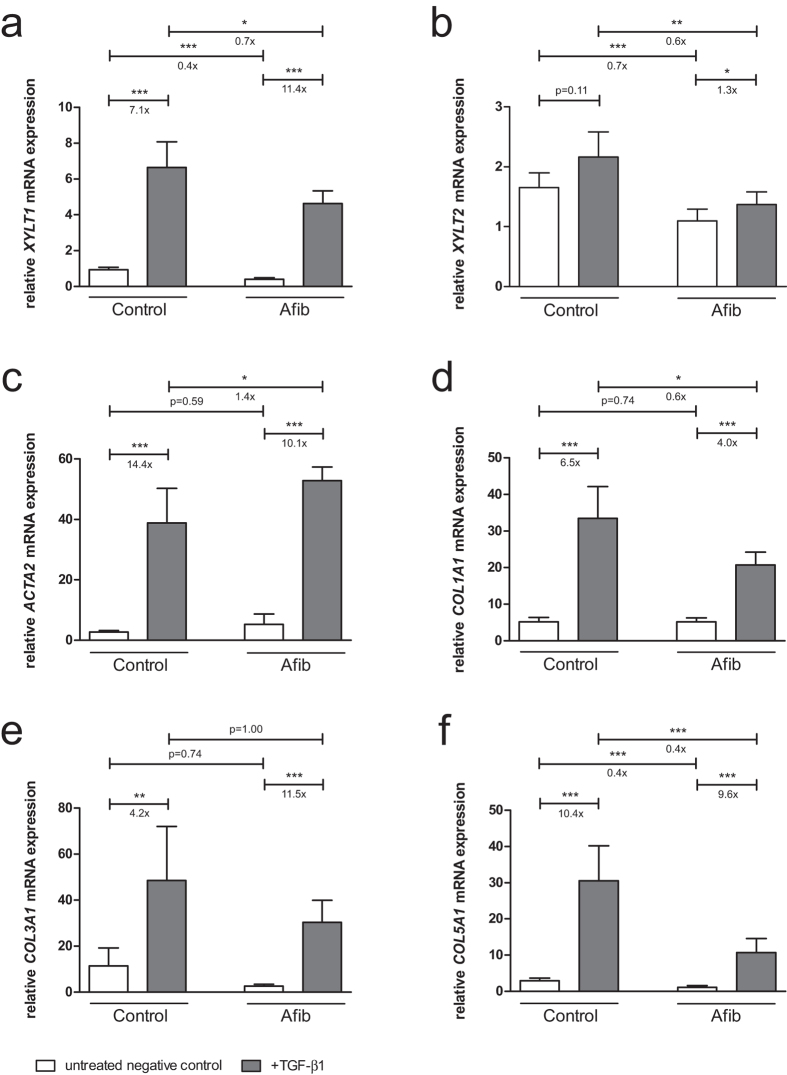
Effect of TGF-β1 on *XYLT* and ECM mRNA expression levels in SF. Control (n = 3) and Afib (n = 6) cell lines of SF were seeded for 24 h. After incubation for another 24 h in serum-depleted medium, SF were treated with TGF-β1 (5 ng/mL; black bars) or vehicle (PBS; white bars) for 48 h. Relative mRNA expression levels of *XYLT1* (**a**), *XYLT2* (**b**), *ACTA2* (**c**), *COL1A1* (**d**), *COL3A1* (**e**), and *COL5A1* (**f**) were analyzed by quantitative real-time PCR. Data were normalized to a normalization factor, determined by calculating the geometric mean of *HPRT*, *GAPDH* and *B2M* mRNA expression levels, and expressed as a ratio to one control cell line. Values are means ± 95% CI. *p < 0.05; **p < 0.01; ***p < 0.001 (Mann-Whitney U-test).

To investigate differences in fibrotic remodeling, control and Afib SF were treated with profibrotic TGF-β1 for 48 h. mRNA expression levels were determined by quantitative real-time PCR. After cytokine supplementation, *XYLT1* mRNA expression was significantly upregulated. The effect was stronger in the Afib than in control cells (11.4- vs. 7.1-fold upregulation). Our analysis revealed that the *XYLT1* mRNA expression level was decreased in Afib in comparison to control SF (Fig. 1a).

A lower *XYLT2* mRNA expression than in controls was also observed in Afib cells, whereas incubation with TGF-β1 was followed by a slight increase in *XYLT2* mRNA expression of control and Afib SF (Fig. 1b, not significant vs. 1.3-fold upregulation). The mRNA expression level of the myofibroblast marker *ACTA2* showed both a slight but not significant increase in the basal expression level between control and Afib SF and an increase in expression after TGF-β1 treatment (Fig. 1c, 14.4- vs. 10.1-fold upregulation). The mRNA expression of all other targets examined in this study was found to be equal or decreased in Afib fibroblasts in comparison to controls. Further analysis revealed a greater increase in *COL3A1* (Fig. 1e, 11.5- vs. 4.2-fold upregulation) and a minor increase in *COL1A1* (Fig. 1d, 4.0- vs. 6.5-fold upregulation) and *COL5A1* (Fig. 1f, 9.6- vs. 10.4-fold upregulation) mRNA expression in Afib vs. control SF after TGF-β1 treatment.

### Effects of TGF-β1 stimulation on XT activity and α-SMA protein expression in SF

**Figure 2:**
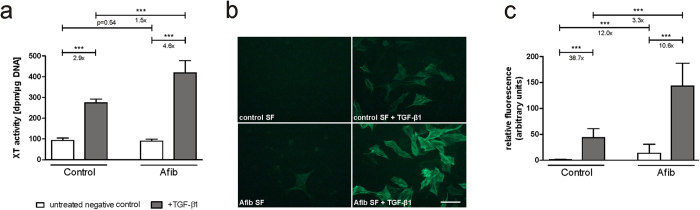
Effect of TGF-β1 on XT activity and α-SMA protein expression in SF. Control (n = 3) and Afib (n = 6) cell lines of SF were seeded for 24 h. After incubation for another 24 h in serum-depleted medium, SF were treated with TGF-β1 (5 ng/mL; black bars) or vehicle (PBS; white bars). After 48 h, XT activity was determined in cell culture supernatants by radiochemical enzyme assay (**a**). After an incubation time of 120 h, α-SMA protein expression was analyzed and quantified by immunohistochemistry (**b**). Values are means ± 95% CI. *p < 0.05; **p < 0.01; ***p < 0.001 (Mann-Whitney U-test). Scale bar: 100 μm.

Changes in the extent of SF myofibroblast differentiation after treatment with TGF-β1 were evaluated by analyzing XT activity in cell culture supernatants as well by immunohistochemical staining of α-SMA. Untreated control and Afib fibroblasts had similar levels of XT activity. After supplementation of TGF-β1, XT activity increased to a greater extent in Afib SF than in controls (Fig. 2a, 4.6- vs. 2.9-fold upregulation). The extent of stimulated XT activity was significantly higher in Afib than in control cells. α-SMA protein expression also increased to a greater extent in Afib than control SF (Fig. 2b,c).

### Determination of serum XT activity

**Figure 3:**
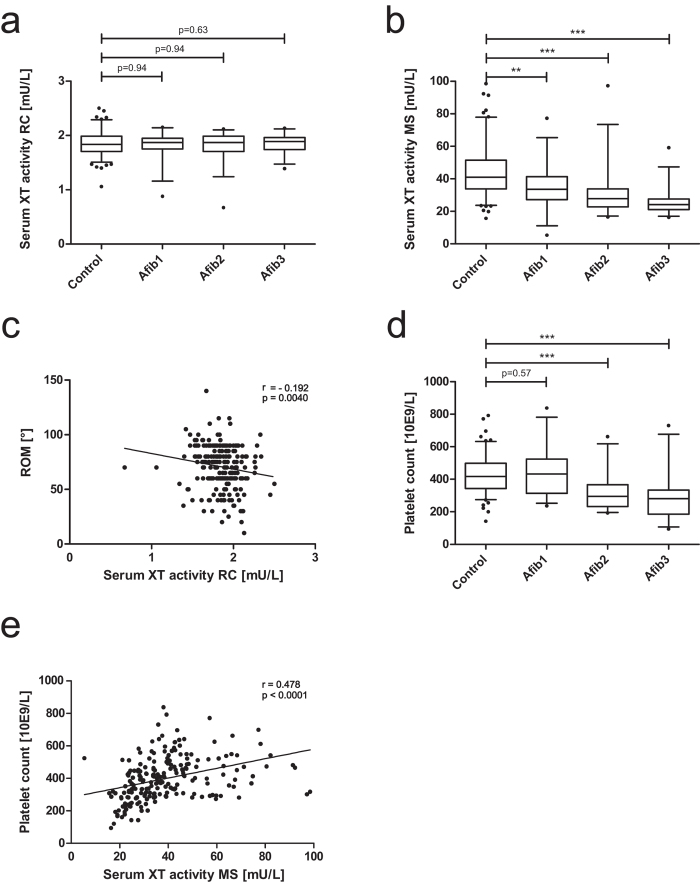
Analysis of XT activity and platelet count in serum of control and Afib patients. Serum XT activity was analyzed in control (n = 132) and Afib patients (n = 95, Afib1 to Afib3: increasing arthrofibrosis score) by radiochemical enzymatic assay: (**a**) mainly depicting XT-I activity or HPLC-ESI-MS based technique, (**b**) mainly depicting XT-II activity. XT activity was correlated to ROM (**c**) as well as platelet count (**d** and **e**). Boxes show the 25^th^ to 75^th^ p**e**rcentiles; horizontal lines in the boxes show the median while vertical whiskers show the 5^th^ to 95^th^ percentile. *p < 0.05; **p < 0.01; ***p < 0.001 (Mann-Whitney U-test).

To determine the suitability of serum XT activity as a biomarker, serum XT activity from Afib and control patients was measured by a radiochemical enzymatic assay (RC) and a HPLC-ESI-MS (MS) activity assay. The use of different acceptor proteins entails divergent substrate conversion rates of XT isoenzymes. The radiochemical XT activity assay (Fig. 3a) mainly reflects XT-I activity, while the HPLC-ESI-MS assay predominantly depicts XT-II activity (Fig. 3b). XT-I activity did not show significant differences between all groups (Fig. 3a). Nevertheless, a significant but weak correlation of serum XT-I activity and the extent of the patient´s range of motion (ROM) was detected (Fig. 3c; r = −0.192, p = 0.004). XT-II activity significantly diminished with an increasing arthrofibrosis score (Fig. 3b). Interestingly, the decrease in XT-II activity correlated with a significant decrease in platelet count (Fig. 3d,e; r = 0.478, p < 0.0001). The occurrence of OA is indicated (Table 1), but did not significantly influence serum XT activity.

### Comparative analysis of galectin-3 and GDF-15 serum levels

**Figure 4:**
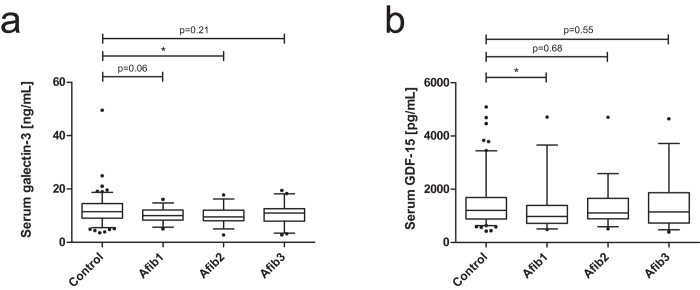
Analysis of serum levels of galectin-3 and GDF-15 in control and Afib patients. Serum concentrations of galectin-3 (**a**) and GDF-15 (**b**) were quantified in control (n = 132) and arthrofibrosis patients (n = 95, Afib1 to Afibf3: increasing arthrofibrosis score) by ELISA. Boxes show the 25^th^ to 75^th^ percentiles; horizontal lines in the boxes show the median while vertical whiskers show the 5^th^ to 95^th^ percentile. *p < 0.05; **p < 0.01; ***p < 0.001 (Mann-Whitney U-test).

To analyze whether common fibrosis-associated parameters are changed by arthrofibrosis, serum galectin-3 and GDF-15 levels were quantified. In comparison to controls, serum galectin-3 as well as GDF-15 concentrations of Afib patients were not strongly affected (Fig. 4).

## Discussion

In this study, we investigated the role of XT in arthrofibrotic remodeling after knee replacement therapy. There is no standardized clinical definition of arthrofibrosis and no recommended therapeutic strategy. Despite numerous attempts to uncover the pathogenesis, none of the discussed hypotheses is fully accepted. Therefore, novel approaches are essential^3^,^4^. XT catalyze the synthesis and accumulation of proteoglycans in wound healing and fibrosis. XT regulation has never been thought to be affected in arthrofibrosis. Since XT act as fibrotic mediators and participate in the development of joint disorders, it appears to be sensible to examine XT regulation in arthrofibrosis.

To define the role of XT in arthrofibrosis, we exposed control and Afib SF to the XT-I-inducing fibrotic elicitor TGF-β1^24^. Recent results have demonstrated that intraarticular injection of TGF-β1 is followed by synovial membrane hyperplasia in arthrofibrosis^25^. Generally, fibrosis is characterized by an aberrant expression of the multifunctional cytokine TGF-β1 and subsequent stimulation of myofibroblast differentiation. Myofibroblasts are mechano-responsive and highly contractile matrix producing cells characterized by *de novo* expression of α-SMA^26^,^27^,^28^. Recently, human XT-I was found to be a myofibroblast biomarker in skin fibrosis^20^.

We detected a stronger increase in basal α-SMA protein expression as well as in α-SMA protein expression after TGF-β1 treatment in Afib versus control SF. The strongest *ACTA2* mRNA expression level was detected after profibrotic stimulation of Afib SF and significantly differed from *ACTA2* mRNA expression of TGF-β1 induced controls. These findings agree with the histopathological observations by Unterhauser *et al*., who found α-SMA positive fibroblasts in arthrofibrotic tissues^29^. Importantly, we also found a stronger increase of *XYLT1* mRNA expression level in Afib than control SF after TGF-β1 treatment. Hence, XT-I participates in arthrofibrotic myofibroblast differentiation and manifestation. *XYLT2* mRNA expression levels increased only slightly. A lack of contribution of XT-II in fibrosis concurs with the results of earlier studies on cardiac or skin fibrosis^20^,^24^. Basal XT activity did not differ between control and Afib SF, whereby the latter showed a stronger induction of XT activity after TGF-β1 supplementation reinforcing cellular modulation of arthrofibrotic remodeling by XT-I. So the highest XT activity was detected after TGF-β1 treatment of Afib cells. Due to the low basal *XYLT1* mRNA expression in Afib cells, this observation did not completely reflect mRNA expression levels. It is at present unclear how expression of *XYLT1* is regulated on mRNA, protein and enzymatic activity level in arthrofibrosis. There might be higher *XYLT1* mRNA stability, increased translation or increased enzyme activity in Afib fibroblasts which suppress transcription of *XYLT1*. Further studies are needed to unravel regulation of *XYLT1* mRNA and protein expression in arthrofibrosis. But, due to the lack of specific antibodies, we were not able to perform a protein quantification of human XT-I or XT-II, e.g. by Western blot analysis.

Previous investigations have also shown that TGF-β1 is capable of stimulating dermal collagen formation^30^. Arthrofibrotic remodeling is equally characterized by an increased expression of different collagens^8^,^31^. Our data on the mRNA expression levels of *COL1A1*, *COL3A1* and *COL5A1* confirm that collagen synthesis is stimulated in arthrofibrosis. A stronger upregulation of *COL3A1* and a weaker increase in the *COL1A1* respectively *COL5A1* mRNA expression level was detected in Afib vs. control SF. The mRNA expression levels of all genes discussed here, except *ACTA2*, depicted a minor or not significantly different value of, basal or TGF-β1 induced, mRNA expression in Afib than in control cells. This observation has never been described before, but may be caused by differences in the complex regulation system of mRNA stability. The effects should be further validated on protein level.

In the second part of this study, we analyzed serum XT activity of Afib patients in comparison with controls by performing two enzymatic assays employing different acceptor proteins^22^,^23^. Based on aberrant substrate affinities, enzymatic activities of the XT isoenzymes can be distinguished tendentially^32^,^33^. Analyses did not reveal differences in XT-I activity, although a weak but statistically significant correlation of XT-I activity and the extent of patient´s ROM was detected. In conclusion, a diminished ROM was slightly associated with elevated levels of serum XT-I activity. XT-II activity showed a significant decrease with increasing Afib score. Compared to the current literature, these results are unexpected because fibrosis is known to upregulate serum XT activity^15^,^16^. Recently, Condac *et al*. engineered a *XYLT2* knockout mouse and demonstrated that XT-II is the predominant serum isoenzyme. It could be shown that XT-II is synthesized in platelets^34^. Besides, XT-II is also expressed in every human cell type. But, as mentioned above, XT-II is not involved in fibrotic remodeling of the ECM. Thus, an increase in serum XT activity in fibrosis is most probably based on an increase in *XYLT1* expression of resident cell as for instance fibroblasts. In this context, it has to be emphasized that arthrofibrosis is a local disorder. Systemic disorders such as scleroderma are characterized by numerous locations of XT-I expression, resulting in increased serum XT activity^16^. Hepatic fibrosis is also characterized by increased serum XT activity due to rapid XT distribution in blood^15^. In OA patients on the other hand, only a minor impairment could be detected in serum XT activity despite the generalized character of OA^35^,^36^.

Therefore, we hypothesize that XT-I activity alterations due to arthrofibrosis affecting a single joint are too small to be detected, even though the correlation of serum XT-I activity and the ROM gives a weak hint to a relationship between arthrofibrosis and increased serum XT-I activity levels. A local change in XT expression level will lead to altered synovial fluid XT activity. But a subsequent increase in serum XT activity would require XT to pass through the blood-joint barrier^37^. The slight increase in serum XT activity in association with significant increases in the XT activity in synovial fluid in generalized OA confirms the assumption that XT does not overcome the barrier^35^. Measuring XT activity in the synovial fluid of arthrofibrosis patients would be helpful, but the high risk of infection after puncture of the knee joint should be taken into account. On the basis of the indicated hypotheses, we expect a strong increase in XT-I activity in synovial fluid of Afib patients in comparison to controls as well as a stronger negative correlation of the ROM and XT-I activity in the synovial fluid. However, synovial fluid was not available for the study cohort investigated here.

**Figure 5:**
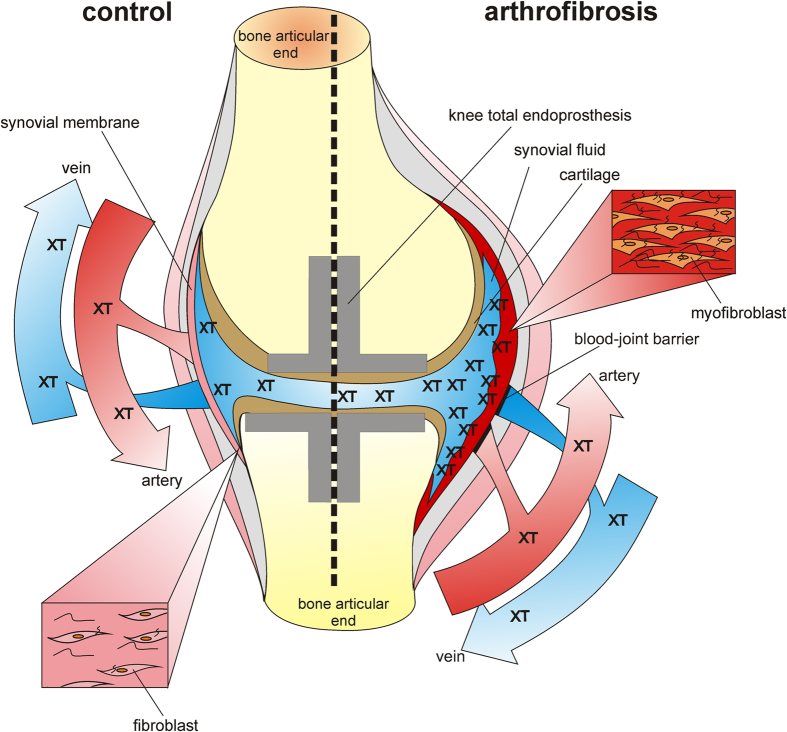
Hypothetical model of arthrofibrosis (drawn by Dr. Isabel Faust). Comparative illustration of the synovial membrane and the fibroblast phenotype in a control or arthrofibrotic knee joint. Due to semi-permeability of the synovial membrane, XT accumulates in the synovial fluid, while serum XT activity remains constant in arthrofibrosis.

We conclude that neither an increase in serum XT activity levels nor an increase in common fibrosis markers is detectable in arthrofibrosis (Fig. 5). We underline this statement by our data on serum galectin-3 and GDF-15 levels. The increase in both markers is associated with scleroderma, OA, and RA^38^,^39^. In this study, no interference of these markers was registered.

We present a correlation between a decrease of human serum XT-II activity in Afib patients and a decreased platelet count. XT-II is not involved in fibrotic remodeling, but its decreased activity might reflect a minor secretion from platelets which in turn gives a hint to thrombocytopenia of Afib patients. Thrombocytopenia has already been described in liver fibrosis. Originally it was thought that a decrease in the platelet count is attributed to the sequestration and destruction of platelets in the enlarged spleen observed in chronic viral hepatitis. But the degree of thrombocytopenia is also associated with a higher grade of fibrosis in chronic viral hepatitis not characterized by splenomegaly^40^. Recently, Kodama *et al*. demonstrated that platelets have an antifibrotic role by suppressing *COL1A1* expression of hepatic stellate cells so that, in a mouse model, thrombocytopenia exacerbated cholestasis-induced liver fibrosis^41^. Nevertheless, the medical reason for thrombocytopenia in liver fibrosis remains elusive.

The role of thrombocytopenia in arthrofibrosis was firstly described and discussed in this study. Theoretically, a decrease in the platelet count might be caused by augmented platelet activation and aggregation. An ongoing platelet activation was also observed in scleroderma and results in elevated serum levels of platelet-derived molecules as for instance platelet factor 4 or serotonin^42^,^43^. Dees *et al*., recently reported a link between vascular disease of scleroderma and tissue fibrosis via platelet-derived serotonin^44^.

Platelets are also potent immune cells regulating pathogenesis of RA. During RA, activated platelets shed microparticles, which are abundant in synovial fluid. The secretion of inflammatory substances as for instance IL-1 of microparticles was shown in RA and emphasizes platelet contribution to immunomodulation^45^,^46^,^47^. Bosch *et al*. already described a chronic inflammatory reaction with infiltration by lymphocytes and plasma cells as well as hypervascularity in arthrofibrotic tissues, although its persistence remains unclear^3^,^48^. In future studies, quantification of mRNA and protein levels should also be used to clarify the involvement of inflammatory factors in development and maintenance of arthrofibrosis. Nevertheless, it is unclear how platelets invade synovial fluid. Both a transportation by leucocytes and a passive efflux across gaps between endothelial cells are discussed^49^,^50^. Therefore, future studies concerning arthrofibrosis should examine whether thrombocytopenia is based on increased microparticle formation. Possibly, the microparticles are sequestered from blood towards the synovial fluid so that serum XT-II activity decreases, while XT-I activity and platelet/microparticle counts increase in the synovial fluid of Afib patients.

In summary, this is the first study showing that arthrofibrosis is a local fibrotic disease. Afib SF were shown to possess the capability of myofibroblast differentiation as shown by upregulated *XYLT1* mRNA expression and XT activity as well as increased *ACTA2* mRNA and protein expression in response to TGF-β1. In contrast, the impact of arthrofibrosis on serum XT activity or other fibrotic serum markers *in vivo* did not reflect the increase in XT activity *in vitro*. The failure to detect arthrofibrosis by its XT activity also applies to other fibrotic serum markers. It appears to be impossible to establish a clinical serum marker for arthrofibrosis of a single joint because of the semipermeability of the synovial membrane. Future studies on monitoring arthrofibrosis should therefore rely on local parameters. Efforts to locally inhibit increased XT activity by targeted inhibition could be of great value for the development of an anti-arthrofibrotic therapy.

## Additional Information

**How to cite this article**: Faust, I. *et al*. Human xylosyltransferases – mediators of arthrofibrosis? New pathomechanistic insights into arthrofibrotic remodeling after knee replacement therapy. *Sci. Rep*. **5**, 12537; doi: 10.1038/srep12537 (2015).

## Figures and Tables

**Table 1:** Clinical characteristics of patients with arthrofibrosis and control patients^a^.

	Control	Afib1	Afib2	Afib3
**n**	132	28	36	31
**Age (years ± SD)**	62.0 ± 9.6	65.0 ± 11.3	61.4 ± 9.7	63.5 ± 9.3
**Time_diagnosis_ (month ± SD)**	6.7 ± 22.6	2.4 ± 4.6	26.0 ± 48.2	19.2 ± 21.6
**ROM (° ± SD)**	78.4 ± 16.3	59.6 ± 16.8***	59.9 ± 20.8***	60.6 ± 24.7***
**Platelet count (10E9/L ± SD)**	427.8 ± 116.1	449.7 ± 144.2	322.5 ± 116.4***	299.7 ± 147.6***
**XT activity RC (mU/L ± SD)**	1.9 ± 0.2	1.8 ± 0.2	1.8 ± 0.3	1.9 ± 0.2
**XT activity MS (mU/L ± SD)**	44.2 ± 16.4	34.6 ± 13.0**	31.5 ± 15.3***	25.7 ± 8.1***
**OA**	117 (88.6%)	27 (96.4%)	23 (63.9%)	20 (64.6%)
**Posttraumatic arthropathy**	3 (2.3%)	3 (10.7%)	0	0

^a^*Afib* increasing arthrofibrosis score Afib1 to Afib3, *ROM* range of motion, *Time*_*diagnosis*_ time between knee replacement therapy and blood sampling/diagnosis in the rehabilitation center, *XT activity RC* XT activity measured by radiochemical assay, *XT activity MS* XT activity measured by HPLC-ESI-MS assay, *OA* osteoarthritis. *p < 0.05; **p < 0.01; ***p < 0.001 (Mann-Whitney U-Test).

**Table 2:** Primers used for quantitative real-time PCR analysis.

Gene name	Protein name	Primer annotation	5′–3′ sequence
***ACTA2***	α-SMA	E8/1412U18	GACCGAATGCAGAAGGAG
		E9/1580L17	CGGTGGACAATGGAAGG
***B2M***	B2M	E1/84U22	TGTGCTCGCGCTACTCTCTCTT
		E2/200L21	CGGATGGATGAAACCCAGACA
***COL1A1***	COL1A1	E49/3908U17	GATGTGCCACTCTGACT
		E50/4058L15	GGGTTCTTGCTGATG
***COL3A1***	COL3A1	E34-35/2498U18	GTGGTAGCCCTGGTGAGA
		E39/2780L16	GGGGGTCCTGGGTTAC
***COL5A1***	COL5A1	E3/782U18	CGCTCTCCCGTCTTCCTC
		E4/1021L20	CACCCTCAAACACCTCCTCA
***GAPDH***	GAPDH	E2-3/116U18	AGGTCGGAGTCAACGGAT
		E4/338L18	TCCTGGAAGATGGTGATG
***HPRT***	HPRT	E3/311U18	GCTGACCTGCTGGATTAC
		E6/568L18	TGCGACCTTGACCATCTT
***XYLT1***	XT-I	E11/2489U18	ACTGCCGAATTCACACAC
		E11-12/2633L19	GTGCCTCCTCAGGTTTGAT
***XYLT2***	XT-II	E10/2258U18	CCTTGTGCTGCCCTTGAC
		E11/2352L18	GCCCTGGAAACTCTGCTC
